# Comparison of parameters of bone profile and homocysteine in physically active and non-active postmenopausal females

**DOI:** 10.12669/pjms.325.10655

**Published:** 2016

**Authors:** Sundus Tariq, Khalid Parvez Lone, Saba Tariq

**Affiliations:** 1 Dr. Sundus Tariq, MBBS, M.Phil. Assistant Professor of Physiology, University Medical & Dental College, Faisalabad-38000, Pakistan; 2 Dr. Khalid Parvez Lone, M.Sc., M.I. Biol., Ph.D., F.Z.S.P. Prof. & Head of Department of Physiology/Metabolic Disorders, University of Health Sciences, Lahore, Pakistan; 3 Dr. Saba Tariq, MBBS, M.Phil. Assistant Professor of Pharmacology, University Medical & Dental College, Faisalabad-38000, Pakistan

**Keywords:** Homocysteine, Physical activity, Osteoporosis, Bone Mineral Density

## Abstract

**Background and objectives:**

Optimal physical activity is important in attaining a peak bone mass. Physically active women have better bone mineral density and reduce fracture risk as compared to females living a sedentary life. The objective of this study was to compare parameters of bone profile and serum homocysteine levels in physically active and non-active postmenopausal females.

**Methods:**

In this cross sectional study postmenopausal females between 50-70 years of age were recruited and divided into two groups: Physically inactive (n=133) performing light physical activity and Physically active (n=34) performing moderate physical activity. Physical activity (in metabolic equivalents), bone mineral density and serum homocysteine levels were assessed. Spearman’s rho correlation was applied to observe correlations. Two independent sample t test and Mann Whitney U test were applied to compare groups. P-value ≤ 0.05 was taken statistically significant.

**Results:**

Parameters of bone profile were significantly higher and serum homocysteine levels were significantly lower in postmenopausal females performing moderate physical activity as compared to females performing light physical activity. Homocysteine was not significantly related to T-score and Z-score in both groups.

**Conclusion:**

Improving physical activity could be beneficial for improving the quality of bone, decreasing fracture risk and decreasing serum homocysteine levels.

## INTRODUCTION

Osteoporosis is the most common age related bone disorder in humans caused by environmental and genetic factors.^1^ It is a leading cause of fractures in old age, causing pain, affliction, hospitalization, financial burden, poor life quality leading to early death.^2^ Estimates based on a large ultrasound study conducted throughout Pakistan suggested that there are 9.91 million people (7.19 million women, 2.71 million men) with osteoporosis.^3^ During the normal bone mass life cycle, highest bone mass is attained during the 3^rd^ decade of life followed by a slow and then rapid fall in bone mass.^4^ This risk of bone loss increases if people spend a sedentary life style^5^ and have decreased physical activity.^6^ Increased physical activity is important in optimizing the peak bone mass.^7^ But, if a person comes back to sedentary life after living a physically active life then the loss in BMD is more as compared to the age related loss.^8^

The rate of bone loss is increased dramatically in women after menopause.^4^ This loss is attributed to non-modifiable decline in sex steroids that occurs after menopause. But, there is relatively infrequent evidence that physical activity prevents bone loss or increases bone mineral density after menopause. In addition, physical exercise also induces changes in protein and amino acid metabolism. Homocysteine, a sulfur containing amino acid, is identified as a new risk factor that leads to osteoporosis.^9^ Increase levels of homocysteine increases the fracture risk especially in post-menopausal females by causing oxidative damage to the bones, reducing bone quality and increasing bone resorption.^10^,^11^ Several studies have explored the relation of homocysteine with bone health in postmenopausal females,^9^-^12^ but whether physical activity also affects homocysteine levels in postmenopausal females is not completely understood.

The present study was designed to compare parameters of bone profile and homocysteine in physically active and non-active postmenopausal females.

## METHODS

The present cross sectional study was carried out on 167 postmenopausal females grouped into two categories depending on the physical activity scores. Physically inactive females (n=133) performing light physical activity were placed in group A and Physically active females (n=34) performing moderate physical activity were placed in group B. One female performing vigorous physical activity was not included in any group and is also excluded from statistical analysis. Convenient sampling was done and subjects were selected from general population on the basis of inclusion and exclusion criteria. This study was conducted from February 2012 to March 2013. Females within age range of 50 to 70 years with at least three years of amenorrhea were included while females on medications affecting bone mineralization, steroids, cyclosporine, antifolate drugs, oral contraceptives/hormone replacement therapy, multivitamins and bisphosphonate therapy were excluded. Women with renal or liver disease, coronary artery disease, severe psoriasis, systemic diseases like hyperthyroidism, hyperparathyroidism and premature menopause were also excluded from the study.

Ethical review committee has given approval to conduct this study according to Helsinki declaration of human rights. Written informed consent was obtained from all participants and detailed general physical examination was performed. Physical activity was recorded in metabolic equivalents and tests to determine the BMD and serum homocysteine levels were performed at Physiology department of University of Health Sciences, Lahore.

### Physical Activity

Self-reported physical activity questionnaire was used to measure physical activity in metabolic equivalents (METs).^13^ One MET is equivalent to energy required to sit quietly. For an average adult, it is one calorie per every one kilogram of body weight per hour. Physical activity can be classified into light, moderate, and vigorous intensity activity. Slow walk, sit quietly (watching TV, knitting), standing light work (dusting, washing dishes, cooking), fishing while sitting, playing instruments (piano) are included in light activity (less than 3.0 METs). While in moderate activity (3.0 to 6.0 METs) brisk walking at four miles per hour (mph), heavy cleaning (window washing, mopping, vacuuming,), mowing lawn, bicycling—light effort at 10 to 12 mph, games (recreational badminton, tennis doubles) are included. In vigorous activity (more than 6.0 METS) very brisk walking, hiking, carrying heavy load, jogging at 6 mph, shoveling, bicycling fast at 14 to16 mph, games (Tennis singles, soccer and basketball) are included.

### Bone mineral density

BMD of postmenopausal females was assessed from distal metaphysis of the proximal phalanges of fingers II to V (index, middle, ring and little finger) using DBM Sonic Bone profiler manufactured by IGEA, Capri, Italy, Model: BP01. Parameters of bone profile i.e. amplitude dependent speed of sound (ADSOS), T-score, Z-score, ultrasound bone profile index (UBPI) and bone transmission time (BTT) were computed. UBPI is an index of the future fracture risk. It indicates the probability that the patient has an osteoporotic fracture. Its range is from 0 to 1. Lower the value, higher is the probability of fracture.^14^

### Blood sample collection

Five ml fasting blood was collected from antecubital vein. It was dispensed in serum tubes. Serum was separated by centrifugation at a speed of 3000 revolutions per minute (rpm) for 10 minutes. The serum was stored in aliquots at -40°C until used. Serum total homocysteine levels were determined by homocysteine enzyme immunoassay (EIA) manufactured by Axis Shield Diagnostics limited, Dundee United Kingdom, with an automated EIA analyzer (Bio-Rad Laboratories, Hercules, CA, USA). The total run precision was 10%.

### Statistical analysis

The data were entered and analyzed using IBM-SPSS version 20 (Statistical Package for Social Sciences). Normal distribution of the data was checked by Shapiro-Wilk’s statistics and if p-value was ≤ 0.05 data was considered to be non-normally distributed. Frequencies and percentages were given for qualitative variables. Mean **±** SEM was given for normally distributed quantitative variables. Median with IQR was given for non- normally distributed quantitative variables. Spearman’s rho correlation was applied to observe correlations. Mann Whitney U test and independent sample t test were applied to compare two groups. P-value ≤ 0.05 was taken as statistically significant.

## RESULTS

**Fig.1:**
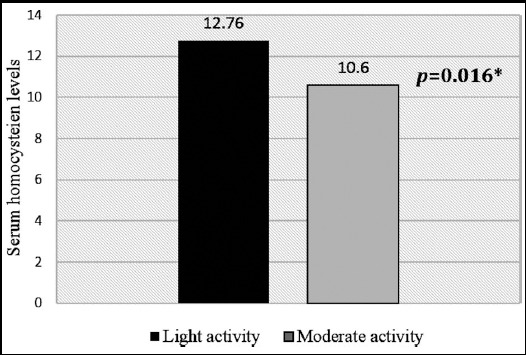
Comparison of serum homocysteine levels in postmenopausal females performing light and moderate physical activity using Man Whitney U test. * p-value ≤ 0.05 is considered statistically significant.

**Fig.2:**
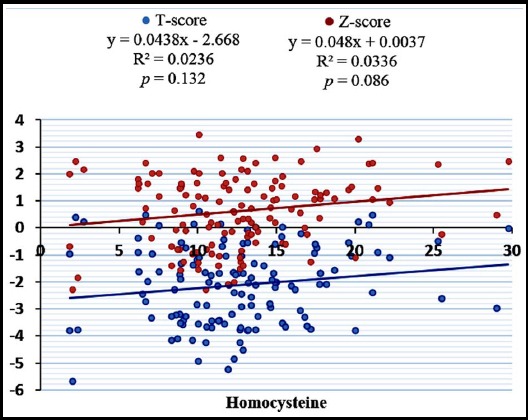
Scatter plot showing correlation of serum Homocysteine with T and Z-score in postmenopausal females performing light physical activity.

**Fig.3:**
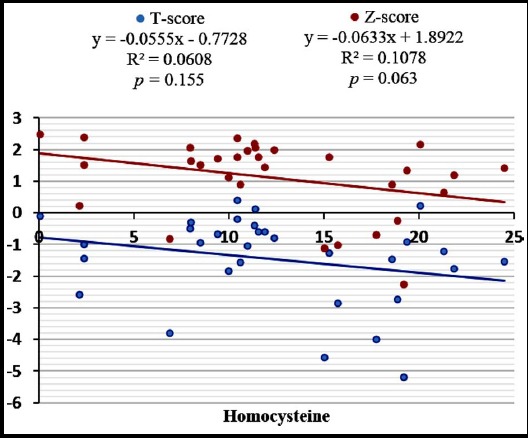
Scatter plot showing correlation of serum Homocysteine with T and Z-score in postmenopausal females performing moderate physical activity.

Postmenopausal females (n= 168) performing light, moderate and vigorous physical activity were 79.2% (n= 133), 20.2% (n= 34) and 0.6% (n= 1) respectively. Female performing vigorous physical activity was not included in statistical analysis. The parameters of bone profile, i.e. ADSOS (*p* = 0.009), T-Score (*p* = 0.009), Z-Score (*p* = 0.041), UBPI (*p* = 0.011) and BTT (*p* = 0.005) were significantly higher in postmenopausal females performing moderate physical activity as compared to females performing light physical activity (Table-I). Serum homocysteine levels were significantly higher in postmenopausal females performing light physical activity as compared to postmenopausal females performing moderate physical activity (Fig.1). Serum homocysteine levels were not significantly related to T-score and Z-score in both groups (Fig.2 & 3).

**Table-I:** Comparison of parameters of bone profile (BMD) with light and moderate physical activity in postmenopausal females using Mann Whitney U test.

*Parameters*	*Light activity n=133*	*Moderate activity n=34*	p-value*
ADSOS	1982 (1884 – 2056)	2049 (1998 – 2085)	0.009
T-Score	-2.02 (-3.41 to -0.96)	-1.07 (-1.80 to -0.55)	0.009
Z-Score	0.75 (-0.38 to 1.60)	1.44 (0.48 – 1.98)	0.041
UBPI	0.37 (0.24 – 0.53)	0.48 (0.35 – 0.61)	0.011
BTT^#^	1.31 ± 0.01	1.43 ± 0.03	0.005

Values are given as Median (IQR).

# Value is given as Mean ± SEM and comparison is done using 2 independent sample t-test

* p-value ≤ 0.05 is considered statistically significant.

## DISCUSSION

Physical activity is an important factor that prevents osteoporosis by increasing mechanical strength of bones and muscles, stimulating bone formation and increasing bone mineral density, but in older individuals it has less mechanical effects on the bone.^8^ In present study, physical activity was significantly related to parameters of bone profile. ADSOS, T-Score, Z-Score, UBPI and BTT were significantly higher in postmenopausal females performing moderate physical activity as compared to postmenopausal females performing light physical activity showing that more a woman was physically active more was the BMD. Ultrasound bone profile index, an indicator of future fracture risk, was significantly higher in postmenopausal females who were physically more active showing that these females have a lower risk of fractures. This positive relation of physical activity with bone mineral density in postmenopausal females has been seen in other studies as well.^15^,^16^ Studies have shown that improved physical activity combined with proper nutrition may help to promote bone mineralization not only in infants^17^ but also in elderly people and is associated with long term beneficial skeletal effects that could possibly reduce fracture risk.^18^,^19^

Other than BMD the mean serum homocysteine levels were significantly low in postmenopausal females performing moderate physical activity as compared to postmenopausal females performing light physical activity which simply means that greater physical activity may reduce homocysteine levels in postmenopausal females. Our results were consistent with another study which found that muscular and skeletal physical activity served to decrease levels of Hcy and this relationship was independent of intake of vitamin supplements, fruits and vegetables. Although nutritional status is important, Hcy levels are also largely modulated by physical activity.^20^ It has been shown in a study that fracture risk was higher in women with Hcy in the highest quartile, but not when adjusted for age. Hcy may be considered a marker of frailty that can be modulated by factors such as nutritional state, physical activity and renal impairment.^21^ So physical activity is important not only for better BMD but also for reducing serum levels of Hcy that may indirectly improve the BMD and exercise programs may modulate serum homocysteine levels in a positive way in postmenopausal females. Homocysteine has also been recognized as a potent thrombogenic compound and it promotes platelet adhesion and stimulates the atherogenic process.^22^ So, exercise could be beneficial in reducing the Hcy levels and thus preventing these harmful effects as well.

Studies have shown that high homocysteine levels reduce the bone mineral density in postmenopausal females leading to osteoporosis and also increases fracture risk. This effect may be due to inhibition of enzyme lysyl oxidase which forms collagen cross links and makes bone matrix stable,^11^ by reducing bone blood flow^23^ or by an increase in osteoblast activity as well as osteoclast activity in response to increased homocysteine levels with predominant osteoclast activity.^24^ In our study, no significant correlation was seen between homocysteine and parameters of bone profile (T-Score, Z-Score) in both physically active and non-active postmenopausal females. Similar to this no such relation has been observed in other studies as well.^25^,^26^ So there is equivocal data available regarding this relation and this may be attributed to the small sample size, differences in genetic or environmental factors.

In Pakistan where osteoporosis is a prevalent condition and is causing a major social and financial burden, simply changing the lifestyle by improving physical activity may help in improving bone health in postmenopausal females and reduces the homocysteine levels whose high levels are a risk factor for osteoporosis.

## CONCLUSIONS

Improving physical activity could be beneficial for improving the quality of bone, decreasing fracture risk and decreasing homocysteine levels. The health related risk, social and financial burden associated with osteoporosis in postmenopausal females can be reduced by simply improving the physical activity.
